# Efficient Classification of White Blood Cell Leukemia with Improved Swarm Optimization of Deep Features

**DOI:** 10.1038/s41598-020-59215-9

**Published:** 2020-02-13

**Authors:** Ahmed T. Sahlol, Philip Kollmannsberger, Ahmed A. Ewees

**Affiliations:** 1 Computer Department, Damietta University, Damietta, Egypt; 2 Center for Computational and Theoretical Biology, University of Würzburg, Würzburg, Germany

**Keywords:** Image processing, Acute lymphocytic leukaemia, Computer science

## Abstract

White Blood Cell (WBC) Leukaemia is caused by excessive production of leukocytes in the bone marrow, and image-based detection of malignant WBCs is important for its detection. Convolutional Neural Networks (CNNs) present the current state-of-the-art for this type of image classification, but their computational cost for training and deployment can be high. We here present an improved hybrid approach for efficient classification of WBC Leukemia. We first extract features from WBC images using VGGNet, a powerful CNN architecture, pre-trained on ImageNet. The extracted features are then filtered using a statistically enhanced Salp Swarm Algorithm (SESSA). This bio-inspired optimization algorithm selects the most relevant features and removes highly correlated and noisy features. We applied the proposed approach to two public WBC Leukemia reference datasets and achieve both high accuracy and reduced computational complexity. The SESSA optimization selected only 1 K out of 25 K features extracted with VGGNet, while improving accuracy at the same time. The results are among the best achieved on these datasets and outperform several convolutional network models. We expect that the combination of CNN feature extraction and SESSA feature optimization could be useful for many other image classification tasks.

## Introduction

Blood contains mainly three cell types: red blood cells, platelets and white blood cells. Red blood cells are important for oxygen transport from the heart to all tissues, and carry away carbon dioxide^1^. They comprise up to 50% of the overall volume of blood. White Blood Cells (WBCs) also have important functions for the immune system, as they are the main defense of the body against infections and diseases^2^. The reliable classification of WBCs is therefore important and increasingly demanded. WBCs can be categorized into two types, defined by the appearance of the cytoplasm. The first type are Granulocytes and include Basophils, Eosinophils and Neutrophils. The second group, called Agranulocytes, includes Lymphocytes and Monocytes. Millions of people are affected by Leukemia, which is considered as a malignant tumor. It starts in the lymphatic system, where blood cells are produced. Firstly, it begins in the bone marrow and is then distributed in the blood cells of the entire body. Normally, WBCs grow based on body needs, but in case of Leukemia, they are created abnormally and become inefficient. Although they can often be detected by their dark purple-like appearance, the analysis and further processing become very complicated due to variability in shape and texture. The category of Leukocytes includes cells that can greatly vary between each other. While they can be distinguished by their shape and size, one challenging aspect is that WBCs are surrounded by other blood components like red blood cells and platelets.

**Figure 1:**
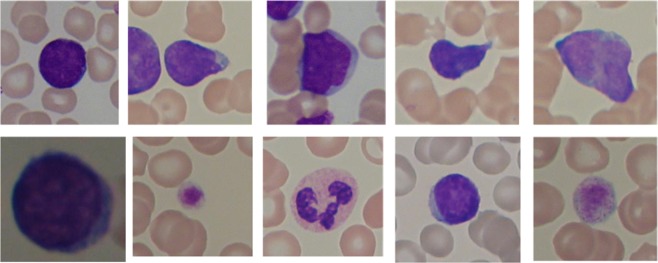
Samples from the ALL-IDB2 dataset^2^ showing benign (top) and malignant (bottom) lymphocytes.

As seen in Fig. 1, lymphocytes have a rather regular shape, their nuclei have smooth and regular edges, whereas lymphocytes from patients with Acute Lymphocytic Leukemia (ALL), so-called lymphoblasts, have a less regular envelope and display small cavities in their cytoplasm, so-called vacuoles, and round particles within their nuclei, so-called nucleoli. As the described changes in morphology get more pronounced, the indication of the disease becomes more severe.

Deep learning using Convolution Neural Networks (CNN)^3,4^ is currently the best choice in medical imaging applications such as detection and classification^5,6^. While CNNs achieve the best results on large data sets, they require a lot of data and computational resources to train. In many cases, the dataset is limited and may not be sufficient to train a CNN from scratch. In such a scenario, in order to leverage the power of CNNs and at the same time reduce the computational costs, transfer learning can be used^7,8^. In this approach, the CNN is initially pre-trained on a large and diverse generic image data set and then applied to a specific task^9^. There are several pre-trained neural networks that have won international competitions like VGGNet^10^, Resnet^11^, Nasnet^12^, Mobilenet^13^, Inception^14^ and Xception^15^. In^16^ an evaluation of different CNN architectures was performed, and transfer learning achieved top-scoring performance on thoraco-abdominal lymph node (LN) as well as interstitial lung disease (ILD) classification. The authors of^17^ used average pooling classification to distinguish malignant from non-malignant cells after they extracted features from breast cancer images using pre-trained CNN architectures fed into a fully connected classification layer. The experimental results showed that the detection accuracy of their model outperforms all other CNN approaches in cytological image-based detection and classification of breast tumors. Other work builds on a combination of multiple deep learning architectures to improve the usefulness of transfer learning for cell-based image classification^18,19^. In^17^, transfer learning was used to overcome limitations of previously published models for breast cancer detection in cytology images on standard benchmark datasets.

These approaches have in common that they use a large number of features (up to 100 K) from pre-trained CNN models. This is inefficient in terms of time and computational resources since many of these features are redundant or contain zeros. Moreover, classifier accuracy can benefit from limiting the number of features. In our previous work^20,21^, detection of white blood cells was performed by extracting different features including color, texture, shape, as well as hybrid features using classical image processing, and then applying a social spider-inspired optimization to choose the most useful features. The model was tested on ALL-IDB2, the same dataset as in this work. The segmentation results were 99.2%, 100% and 97.1% for accuracy, sensitivity and specificity, respectively, and the model classification accuracy was the best published yet.

In this work, a novel approach is proposed to distinguish between benign and malignant WBCs as shown in Fig. 1. The proposed approach combines convolutional neural networks (CNNs) with an improvement of the salp swarm algorithm (SSA) based on statistical operators. A variation of CNN called VGGNet previously trained on millions of images is used for feature extraction. The last layer of VGGNet can be removed so an image can be passed through the rest of the network to obtain its feature vector. This way, the CNN is used to extract a huge feature matrix for each image which can then be passed to an external classifier for image classification. The dataset used in this study has only two classes (benign and malignant), so the model was modified accordingly. The feature matrix produced by the CNN needs to be adjusted to be suitable for image classification. For this reason, we developed a Statistically Enhanced Salp Swarm Algorithm (SESSA) to improve classification performance by excluding correlated and noisy features and selecting only the most relevant features.

The main focus of our manuscript is to present a novel method for image feature selection based on improved swarm optimization and to show that it outperforms many existing approaches for classification of WBCs to detect leukemia. We focus on this application since it is a challenging problem with high medical relevance, for which good benchmark datasets are available. The difficulty in detecting leukemic cells from such images lies in the morphological similarity and subject variability, making the definition of suitable image features a very challenging task. Deep convolutional networks perform well at this task but are not very efficient due to their large (and largely redundant) space of learned features. WBC classification for leukemia detection, therefore, provides the ideal test case for swarm-based optimization of feature selection. We do not present a readily usable clinical tool for leukemia diagnosis but offer a new, efficient method to optimize deep learning-based methods for medical image classification. Such methods will play an increasingly important role in image-based clinical diagnosis in the near future.

## Material and Methods

### Extraction of features using convolutional neural networks

**Figure 2:**
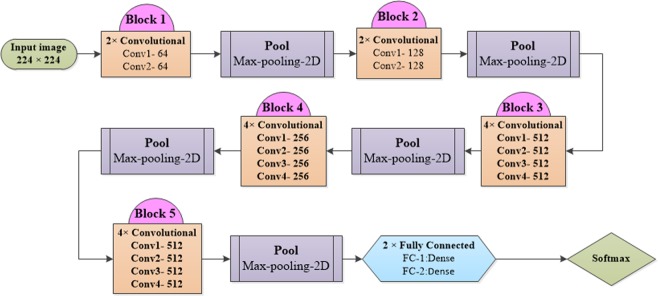
Overview of the VGGNet layer structure (left) and corresponding parameters (right).

The main idea of transfer learning with very deep CNNs is to use a pre-trained deep network previously fit to a big dataset such as ImageNet (ca. 1.2 million images with another 50,000 images for validation and 100,000 images for testing, on 1000 different categories), and adapt it to solve a different image classification problem^22^. As the network already learned relevant image features from a generic training dataset, it has a basis of features that can be used to focus on a particular image type to solve a classification task. In this work, we used a popular and reliable CNN architecture called VGGNet, shown in Fig. 2, with 16 conv (convolutional) and three FC (fully connected) layers. The number of channels (width of the conv layers) is comparably small, from 64 in the initial layer to 512, increasing by a factor of 2 after each max-pooling operation. The input layer has a fixed size of 224 × 224 pixels. As each image is passed through a stack of conv layers, a stride is added to preserve spatial resolution. Pooling is performed by 5 max-pooling layers over a specific window with stride following some but not all conv layers. A stack of conv layers with depth varying in different architectures is followed by three FC layers with 4096 channels in the first two, while the third performs classification^10^. In our case, this layer contains only two channels (one for each class). The final layer is a soft-max layer. All hidden layers have a rectified non-linearity^23^. For each image *X* of study type *T* of the training data, the parameter to be optimized is the weighted binary cross-entropy loss. VGGNet specifications are described in Fig. 2.

Since the shape of the input image is (224, 224, 3), the last layer produced from VGGNet has the shape (7, 7, 512). This means that VGGNet returns a feature vector of 7 × 7 × 512 = 25088 features. In order to perform transfer learning with VGGNet, we first saved the extracted features (bottleneck features) from the pre-trained model, then trained a model (top model) to classify our data using these features, and finally combined our training data and the VGGNet model with the top model to make predictions^4^.

### Salp swarm algorithm

SSA is an optimization method^24^ that imitates the foraging behavior of *Salpidae*, planktonic marine invertebrates. Salps are moving and foraging by a behavior called salp chain, which is an example of swarming behavior. SSA starts by splitting the population into two categories: the front salps, called leaders, and the others, called followers. These salps change their position in order to search for a target (food sources). To perform this movement, Eq. 1 is used to update the position of the leading salps:

1 $${x}_{j}^{1}=(\begin{array}{ll}{F}_{j}+{c}_{1}((u{b}_{j}-l{b}_{j})\times {c}_{2}+l{b}_{j}) & {c}_{3}\le 0\\ {F}_{j}-{c}_{1}((u{b}_{j}-l{b}_{j})\times {c}_{2}+l{b}_{j}) & {c}_{3} > 0\end{array}$$

where ${x}_{j}^{1}$ denotes the leader’s position in *j*-th dimension. ${F}_{j}$ is the target in the dimension. $u{b}_{j}$ and $l{b}_{j}$ are the upper bounds and the lower bounds, respectively. ${c}_{2}$ and ${c}_{3}$ are random numbers in $\mathrm{[0,1]}$. The parameter ${c}_{1}$ is used for balancing between the exploration and the exploitation phases. It is derived using from the following equation:

2 $${c}_{1}=2{e}^{-{(\frac{4t}{{t}_{max}})}^{2}}$$

where the current iteration is *t* of ${t}_{max}$. First, the leaders are updated to change their position, then the followers’ position is updated by the following equation:

3 $${x}_{j}^{i}=\frac{1}{2}({x}_{j}^{i}+{x}_{j}^{i-1})$$

where ${x}_{j}^{i}$ denotes the position of the *i*-th follower and *i* > 1. The main steps of the SSA algorithm are listed in Algorithm 1, adapted from^24^

**Algorithm 1:**
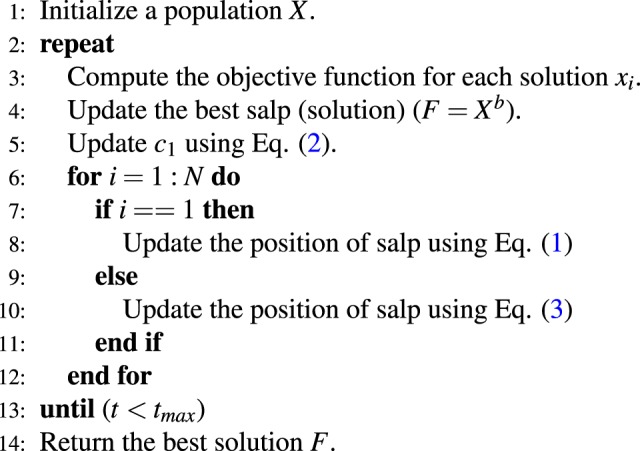
Salp Swarm Algorithm (SSA).

### Feature selection based on SESSA

**Figure 3:**
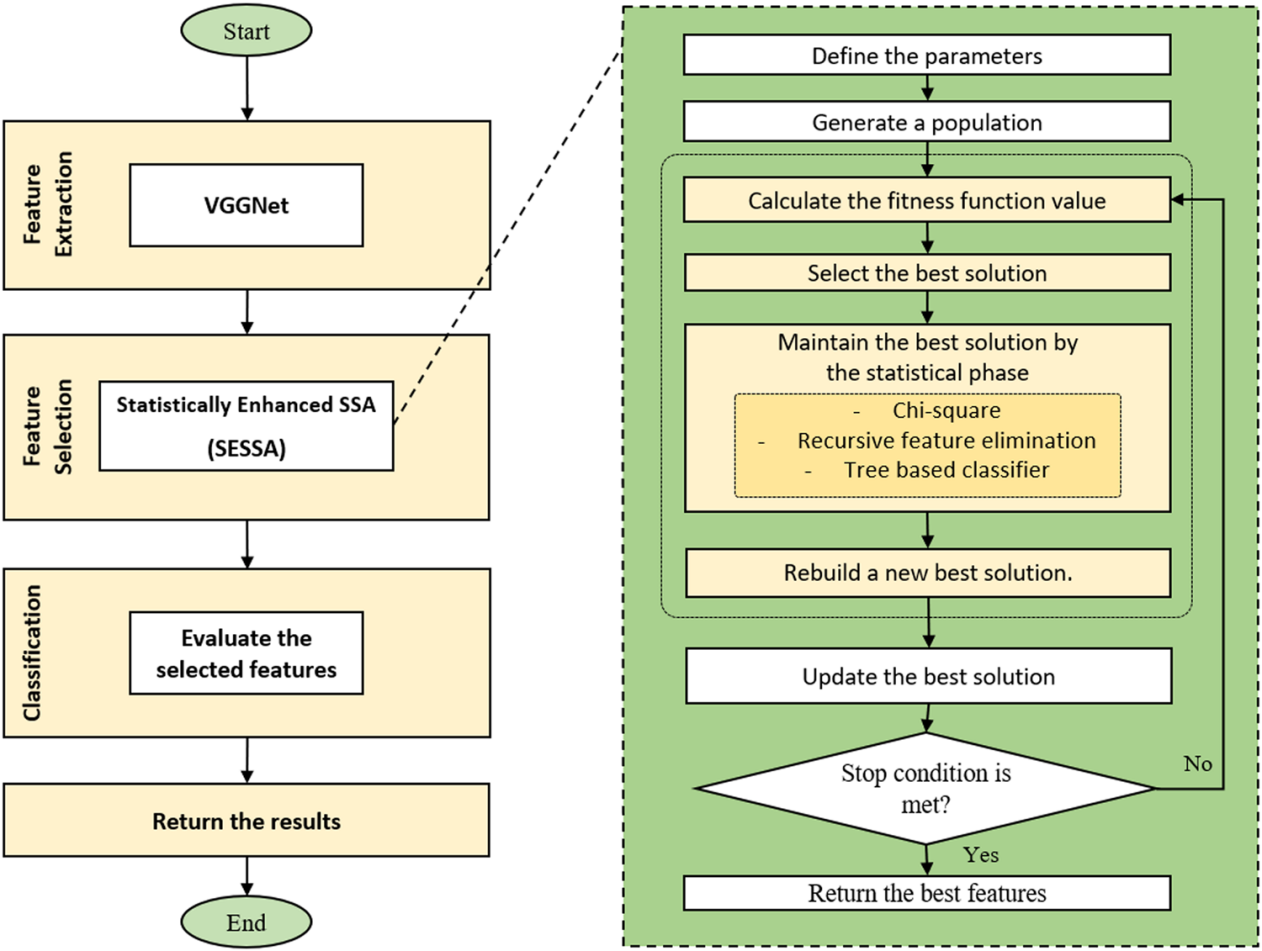
Flow chart of our proposed approach.

After feature extraction using a CNN as described above, we applied feature selection to use only those features for classification that contribute most. Mainly, there are three benefits of performing feature selection - reduced training time (fewer features means that the algorithm trains faster), improved accuracy (less misleading data makes the model more efficient), and reduced over-fitting (higher probability for successful classification). Our new enhanced feature selection method improves the basic SSA by applying statistical operations to exclude irrelevant and noisy features, and by making it more computationally efficient and stable. The overall structure of SESSA is shown in Fig. 3.

The additional operations are as follows:

#### Removing correlated features

Chi-square is used to eliminate correlated features by measuring the dependence between features. Chi-square is computed between each feature for all classes based on (4):

4 $${\chi }^{2}=\mathop{\sum }\limits_{k=1}^{n}\frac{{({O}_{k}-{E}_{k})}^{2}}{{E}_{k}}$$

where *O*_*k*_ = the observed feature value and *E*_*k*_ = the expected feature value. The top k performing ones are then selected as the most relevant features. Subsequently, only the features with the highest score are kept. In this work, several approaches were tested to choose the best k value, which indicates the number of selected features. The higher the number assigned, the larger the proposed model will be. It is not necessarily the case that a higher number of features would improve the model’s performance.

### Recursive feature elimination

This is a greedy optimization approach to find the most efficient subset of features based on a regression model. It chooses the best feature based on coefficients, then sets the feature aside and repeats the process with another set of features. This process is applied until all features in the dataset are exhausted. Finally, features are ranked based on when they were eliminated. The algorithm begins with the full regression model containing all *P* features and then removes the least useful predictor in each iteration. Elimination of features follows these steps: (${\hat{f}}^{P}$ denotes a model with *P* features)

- For each $k=P,\,P-1,\ldots ,1$ remove a feature with the lowest standardized regression coefficient.
- Fit a new model ${\hat{f}}^{P-1}$ and calculate a cross-validated accuracy for classification problem. For regression problems, *AIC*, *BIC* and cross-validated *R*^2^ can be used instead.
- Finally, select the best model from ${\hat{f}}^{P},\,{\hat{f}}^{P-1},\ldots ,{\hat{f}}^{0}$ based on the calculated score values. RFE algorithms selected the best Logistic Regression fit with K-number of manually selected features.

#### Tree-based classifier for feature importance

Tree-based methods are very popular for classification, due to their high level of accuracy and ease of use, as well as robustness. On top of that, they offer two direct methods for selecting features. As known, every individual node in a decision trees is a condition on one feature and splits the set of data into two. This way, similar responses should end up in the same set. The measure to chose the locally optimal condition is termed impurity. During the training of a tree, one can calculate how much each feature reduces the weighted impurity of the tree. Therefore, the impurity decrease can then be averaged per feature, and the features sorted according to their impurity decrease. There is one drawback of this method: when a dataset contains two or more correlated features, there is no preference of one over the other, and any of these features can be used as the predictor. As soon as one of them is selected, however, the importance of the others is immediately reduced, as the impurity they could remove has already been removed by the first feature. This was solved by removing any correlated features from step 1 (removing correlated features).

The fitness function that we used in this study is the root mean square error (RMSE) as in Eq. (5). RMSE is applied to calculate the difference (square error) between the output results and the target for each subset of features. Therefore, a smaller value of RMSE is an indicator of better output results and thus a better feature subset.

5 $$RMSE=\sqrt{\frac{1}{n}\mathop{\sum }\limits_{i=1}^{n}{({y}_{i}-{x}_{i})}^{2}}$$

where, *n* indicates the total number of the set items, *y* and *x* indicate the target data and output data, and $\bar{y}$ indicates the mean of *y*. The number of iterations was set to 100.

### Dataset description

We used two different datasets for this study. The first dataset used in this paper was provided by Department of Information Technology - Universitá degli Studi di Milano^2^. Images were captured with an optical microscope that was coupled to a Canon PowerShot G5 digital camera. The images are provided in JPG format with a color depth of 24 bit. The magnification of the microscope varied between 300 to 500. The ALL-IDB database contains two different datasets, IDB1 and IDB2. We tested our algorithm on the ALL-IDB2 dataset, as it was designed to test the performance of classification systems. This dataset consists of cropped areas of interest of benign and malignant cells from the ALL-IDB1 dataset. These cropped images have similar intensity levels as those in ALL-IDB1, but different image dimensions. This dataset has been used for detection^25,26^, segmentation^27,28^ and classification^29^.

This dataset contains 260 images, 50% benign and 50% malignant. The proposed approach is built for a binary classification problem $y\in \mathrm{0,1}$ because the dataset contains two classes (benign or malignant cell). Figure 1 shows some examples for each class, benign and malignant. It illustrates the variation in cell morphology, structure, shape, and zoom level within the same class on the one hand, and the similarity between images from two different classes on the other hand. Moreover, all images contain other types of blood cells interfering with the white blood cell, whereas some samples contain multiple white blood cells. All these mentioned properties together make the classification task quite challenging.

To overcome the limitation of using a single dataset and to broaden the scope of our work, we extended our study to a second, independent and more recent dataset, C-NMC^30–32^. This dataset was used for the B-ALL normal versus malignant cell classification challenge at IEEE ISBI-2019 and consists of a large number of labeled images of normal and malignant cells. The cell images were extracted from blood smear microscopy images after normalizing the stain, as described in^30–32^. The total size of the training dataset is 10,661 images from 76 subjects. Out of these 7,272 images are from 47 ALL patients, and 3,389 are from 29 normal subjects with healthy cells.

### Validation criteria

To test the performance of the proposed approach, we used accuracy, sensitivity, specificity, precision, F-measure (F1), root mean square error (RMSE), and coefficient of determination (*R*^2^), as well as computational time for selecting features. The definitions of these measures are as follows:

6 $$Accuracy=\frac{{\rm{TP}}+{\rm{TN}}}{{\rm{TP}}+{\rm{TN}}+{\rm{FP}}+{\rm{FN}}}$$

7 $$Sensitivity=\frac{{\rm{TP}}}{{\rm{TP}}+{\rm{FN}}}$$

8 $$Specificity=\frac{{\rm{TN}}}{{\rm{TN}}+{\rm{FP}}}$$

9 $$Precision=\frac{{\rm{TP}}}{{\rm{TP}}+{\rm{FP}}}$$

10 $${F}_{1}=2\times \frac{{\rm{Specificity}}\times {\rm{Sensitivity}}}{{\rm{Specificity}}+{\rm{Sensitivity}}}$$

where “TP” (true positives) refers to the malignant samples that were correctly labeled by the classifier, while “TN” (true negatives) are the benign samples that were correctly labeled by the classifier. “FP” (false positives) are the malignant cells that were incorrectly labeled as benign, while “FN” (false negatives) are the benign samples that were mislabeled as malignant.

11 $$RMSE=\sqrt{\frac{1}{n}\mathop{\sum }\limits_{i\mathrm{=1}}^{n}{({\hat{y}}_{i}-{y}_{i})}^{2}},$$

12 $${R}^{2}\mathrm{=1}-\mathop{\sum }\limits_{i\mathrm{=1}}^{n}\frac{({y}_{i}-{\hat{y}}_{i})}{({y}_{i}-{\bar{y}}_{i})}$$

where ${\hat{y}}_{i}$ denotes the output value, *y*_*i*_ is the target value, and *n* is the samples’ number. ${\bar{y}}_{i}$ is the average of the output values. The datasets were divided into training set and test sets as follows: 80% for training (further split into 80% for training and 20% for internal validation during 5-fold cross-validation) and 20% for testing (external validation). There is no overlap between any of the two sets. For the ALL-IDB2 dataset, these percentages correspond to 208 and 52 images, while for the C-NMC dataset this amounts to 8529 and 2132 images, respectively. Throughout the paper, 5-fold internal cross-validation was applied to all experiments. The number of populations was set to 10, and the maximum number of iterations was set to 100 for each external validation. This strategy was repeated for 30 runs to be able to get an average for our statistical approach. All results are reported on the external test set.

### Implementation environment

The proposed system was implemented in Python 3 on Windows 10 64 bit using a Core i5 CPU and 8 GB RAM. The training was performed on Nvidia Tesla P100 GPU nodes (16 GB GPU memory, 180 GB RAM, 16 vCPUs, Ubuntu Linux 16.04) of the high performance computing cloud *Julia* at the University of Würzburg.

## Results

### Efficiency of the proposed approach

**Figure 4:**
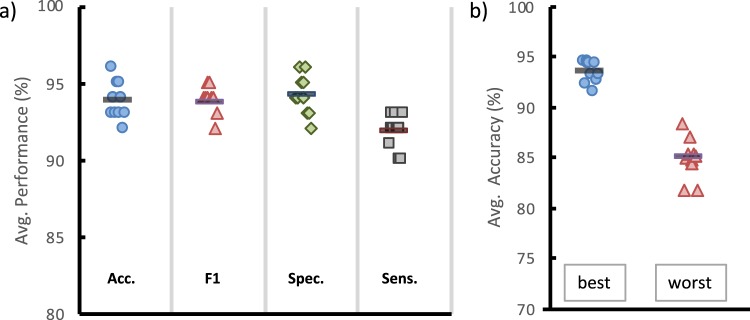
Performance of the proposed hybrid VGGNet and SESSA approach on the ALL-IDB dataset; (**a**) average performance over 10 runs, (**b**) accuracy for 10 best and worst runs.

To evaluate the efficiency of the proposed approach, SESSA was performed in 10 independent runs to produce 10 different feature sets. These sets were evaluated using six classifiers algorithms (Linear SVM, KNN, Decision Trees, Naive Bayes, Adaboost and Multi-Layer Perceptron) which had proven advantageous in our previous works^33–36^. As validation criteria, the mean of the five values (from each fold) was used. Figure 4 shows an average of the 10 feature sets’ performance that was produced by SESSA using accuracy (Acc.), F1, specificity (Spec.) and sensitivity (Sens.) metrics. The results vary between runs due to the nature of the optimization mechanism, which depends on exploring the problem space to search for the best solution.

To demonstrate our method’s reliability, we applied cross-validation for each run produced from SESSA for each classifier and then calculated the average accuracy of the five folds. In Fig. 4, the best and worst classification accuracy were calculated for each of five folds, and the average of all classifiers are reported for each run. Even the worst fold’s accuracy doesn’t go below 80% of classification accuracy, except for the Random Forest classifier, while most of them were close to 90% of classification accuracy.

In Table 1, the extracted features from VGGNet and those extracted from our approach are compared. Only four percent from the extracted features of VGGNet were selected by SESSA. It turns out that the proposed approach which has only about 1 K features achieves better results in most classification criteria than the basic VGGNet feature set which has about 25 K features.

**Table 1:** Comparison of feature number and performance for both datasets.

Dataset 1	Features	Percentage	Accuracy	Specificity	Sensitivity
VGG 19	25088	100%	94.23	**100**	88
Proposed approach	**1087**	**4%**	**96.11**	95	**93**
**Dataset 2**					
VGG 19	25088	100%	80.9	**80.9**	80.9
Proposed approach	**1115**	**4.4%**	**83.3**	67.3	**91.1**

### Efficiency of SESSA feature selection

In this section, four other optimization algorithms are compared to SESSA, namely Statistically Enhanced Multi-verse Optimization (SEMVO), Statistically Enhanced Grey Wolf Optimization (SEGWO), Statistically Enhanced Particle Swarm Optimization (SEPSO) and Statistically Enhanced Genetic Algorithm (SEGA). For a fair comparison, all these algorithms were combined with the same statistical operations to check the effectiveness of both operations and algorithms. Six performance measures are used to evaluate the quality of the produced sub-features, namely RMSE, accuracy, sensitivity, specificity, precision, and *R*^2^. The results of this comparison are shown in Table 2.

**Table 2:** Results of the feature selection compared to other swarm based optimization algorithms for both datasets.

	Alg.	F. no.	Internal validation						Testing (external validation)					
			RMSE	Acc.	Sens.	Spec.	Prec.	F1	RMSE	Acc.	Sens.	Spec.	Prec.	F1
Dataset 1	SESSA	**1087**	**0.108**	**0.985**	**1.00**	**0.969**	**0.971**	**0.985**	**0.1853**	**0.9611**	**0.9955**	**0.9292**	**0.9343**	**0.9622**
(ALL-IDB2)	SEMVO	1121	0.122	0.981	**1.00**	0.961	0.963	0.981	0.1902	0.9610	0.9947	0.9268	0.9304	0.9617
	SEGWO	1101	0.170	0.968	0.999	0.938	0.939	0.967	0.1941	0.9576	0.9942	0.9199	0.9258	0.9587
	SEPSO	1163	0.132	0.979	**1.00**	0.957	0.96	0.979	0.1944	0.9609	0.9929	0.9263	0.9298	0.9615
	SEGA	1158	0.175	0.965	0.997	0.933	0.937	0.966	0.204	0.9547	0.9918	0.9184	0.9247	0.9561
Dataset 2	SESSA	**1115**	**0.382**	**0.854**	**0.923**	**0.700**	**0.872**	**0.897**	**0.409**	**0.833**	**0.911**	0.673	0.85	**0.879**
(C-NMC)	SEMVO	1168	0.419	0.825	0.902	0.662	0.848	0.874	0.447	0.800	0.871	0.645	0.843	0.857
	SEGWO	766	0.407	0.834	0.906	0.676	0.861	0.883	0.427	0.818	0.906	0.634	0.837	0.870
	SEPSO	1196	0.399	0.841	0.916	0.673	0.862	0.888	0.418	0.825	0.897	**0.676**	**0.852**	0.874
	SEGA	1102	0.42	0.824	0.901	0.662	0.848	0.874	0.443	0.804	0.878	0.642	0.842	0.860

On the first dataset (ALL-IDB2), SESSA has the lowest classification error based on the results of RMSE, and SEMVO is on the second place. In addition, SESSA also achieved the highest accuracy, sensitivity, specificity, and precision which indicates that SESSA is able to select higher-quality features than other algorithms. The results of *R*^2^, as a statistical measure, indicate as well that SESSA is the most suitable algorithm and its sub-features are better than the others, followed by SEMVO. In addition, Table 2 shows the computation time along with the best sub-features obtained and the reduction ratio of all algorithms. According to this table, SESSA produces the smallest number of sub-features equal to 1087 with the highest reduction ratio (i.e, 48% of all features) whereas the computational time of SESSA is ranked third after SEGWO and SEGA. Although SESSA is not the fastest algorithm, it produced the highest quality sub-features and was able to reduce the size of the problem to the smallest ratio. Table 3 shows the parameter settings of all algorithms that were applied in all experiments. These settings were taken from the original reference of each algorithm.

**Table 3:** Parameters setting of all optimization algorithms.

Algorithm	Parameters values
SESSA	*C*_2_ ∈ [0, 1], *C*_3_ ∈ [0, 1]
SEMVO	$WE{P}_{min}=0.2$, $WE{P}_{max}=1$
SEGWO	$a\in \mathrm{[2,0]}$
SEPSO	$w\mathrm{=1,}\,wDamp\mathrm{=0.99,}\,C\mathrm{1=1,}\,C\mathrm{2=2}$
SEGA	$pc\mathrm{=0.8,}\,gamma\mathrm{=0.2,}\,pm\mathrm{=0.3,}\,mu\mathrm{=0.02,}\,beta\mathrm{=8}$

On the second, much larger dataset (C-NMC), SESSA still outperforms most of the other feature selection methods, with the only exception of SEPSO showing marginally better specificity and precision. Overall, the performance in all cases is much lower on the new dataset compared to ALL-IDB2. Upon closer investigation, we found that for some subjects (ID H36, H29, H35, H50, H34, H25 and H33) the accuracy is below 0.6, and in particular for H25 (which contains only 19 samples) it is only 0.2. Most of these subjects are ALL patients, same as the accuracy in the class level^37^.

**Figure 5:**
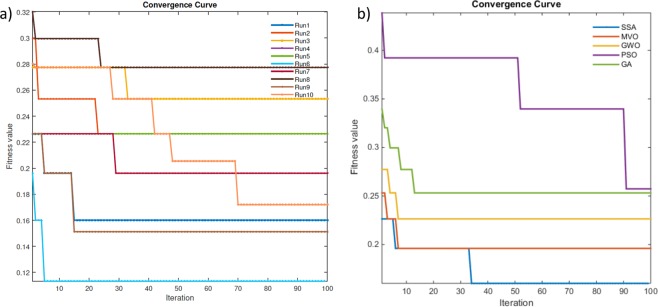
Convergence curves of the proposed approach and of other optimization approaches, (**a**) for 10 independent runs of SESSA, and (**b**) compared to other algorithms.

The convergence behaviour of SESSA was evaluated over ten independent runs, and the convergence curves are shown in Fig. 5a. In this figure, the x-axis represents the iterations while the y-axis represents the fitness value. In addition, the convergence curves of SESSA along with the curves obtained by the compared algorithms are illustrated in Fig. 5b, showing that SESSA exhibits a faster convergence than the other optimization algorithms and obtained the best fitness value after only 34 iterations.

### Comparison with other CNN architectures and related works

In this subsection, the performance of the proposed approach is compared to other convolutional neural networks in terms of classification accuracy and time consumption. It should be noted that all compared deep neural networks are more complex than the proposed approach in terms of structure and consequently, the feature set produced. For example, Nasnet^12^ produces 487 K features, Resnet^11^ and Xception^15^ produce 100 K features, Inception^14^ produces 51 K features, while Mobilenet^13^ produces 50 K features, compared to VGGNet which produces 25 K features.

**Figure 6:**
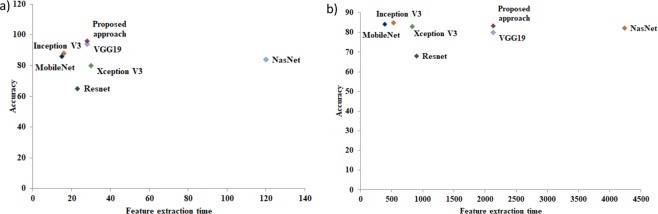
Feature extraction time and accuracy on the ALL-IDB2 dataset (**a**) and on the C-NMC dataset (**b**) compared to other CNN models.

From Fig. 6 (left), it can be seen that on the ALL-IDB2 dataset, our proposed approach outperforms other deep convolutional neural network models like Resnet, Xception, Mobilenet, Nasnet, with a slight advantage over VGGNet. It also shows that the proposed method can extract the least number of features, which means better performance with less resource consumption and efficient use of storage capacity. While feature extraction time was among the smallest in our hybrid model, it was larger than for some of the other deep networks. This is because VGGNet is more complex, as there are more weight parameters (550 MB weight size) resulting in longer inference time. For the much larger and more challenging C-NMC dataset (Fig. 6, right), our model still shows an overall accuracy of 83.2%, putting it third after MobileNet (84.9%) and Inception (84.2%).

In Table 4, a comparison with related feature extraction work on both datasets is shown. Although the classification accuracy we previously reported in^21^ on the ALL-IDB2 dataset reached 95.67% with a spider optimization algorithm for feature selection, it required complex preprocessing operations on the raw images, including noise removal and several segmentation steps. We also had used hand-crafted features which is tedious and time consuming. Instead, our new hybrid approach proposed here works fully automated on the raw images, with no preprocessing or manual steps required. Other previous work also used hand-crafted features, which take a long time to generate - not to mention potential problems with compatibility of features with each other, such as features with different dimensions or features that require a specific image type.

**Table 4:** Comparison with related works on ALL-IDB2 (top) and C-NMC (bottom).

Dataset 1	Features	Classifier	Feature extraction	Accuracy %
Singhal *et al*.^40^	Texture	SVM	Manual	89.72
Singhal *et al*.^41^	Texture	KNN	Manual	93.84
Bhattacharjee *et al*.^42^	Shape	KNN	Manual	95.23
Sahlol *et al*.^21^	Shape, color, texture	KNN	Manual	95.67
Proposed approach	Deep features (VGG19)	SVM	Autom.	**96.11**
**Dataset 2**	**Features**	**Classifier**	**Feature extraction**	**F1%**
Marzahl *et al*.^43^	Deep features (ResNet 18)	CNN	Autom.	86.9
Ding *et al*.^37^	Deep features (various)	CNN	Autom.	86.7
Kulhalli *et al*.^44^	Deep features (ResNeXt)	CNN	Autom.	85.7
Proposed approach	Deep features (VGG19)	SVM	Autom.	**87.9**

## Discussion

The hybrid approach we present here successfully combines two important targets of machine learning: high accuracy and small feature number. This also implies faster computation time and lower resource consumption, which both become increasingly relevant. We believe that reducing the size of the feature vector from 25 K as extracted from VGGNet to about 1 K after SESSA optimization while improving performance at the same time can be considered a successful improvement of a machine learning approach. Our results agree with other related work^38^ where the top-performing models for image classification were ResNet and VGGNet rather than other convolutional neural network architectures. The best pre-trained visual feature extractor in several experiments so far was reported by Kornblith *et al*.^39^.

Using only 208 (80%) samples for training VGGNet while retaining the other 52 samples (20%) for testing the model’s performance proved to be challenging because deep learning models need large amounts of data to generate precise weights, and consequently, to work efficiently. Enhancing the SSA algorithm by adding statistical operations positively affected the performance because it reduces the selected features set by selecting only the best features. The statistical operations applied to SSA evaluate each feature and keep only the most relevant ones. These steps led to preserving only 10% of the original features, which consequently reduces the running time. In addition, the higher accuracy obtained by the proposed algorithm compared to other algorithms can be due to several advantages of SESSA for optimization tasks such as fast convergence, the ability to balance between exploration and exploitation phases, and the ability to escape from local optima. On top of that, it is easy to implement and has only few parameters.

Using optimization algorithms for feature selection shows great potential for complex classification tasks, which might otherwise require days to train a model. This approach can save power and resource consumption while at the same time boosting performance. Moreover, it is not necessarily the case that deeper models perform better, as evident from our comparison with highly complex models such as NasNet and Mobilenet. Instead, choosing the model architecture that best fits the problem can positively affect performance.

## Conclusion

In this work, a hybrid classification approach for White Blood Cell Leukaemia image classification was proposed. It is based on using a deep convolutional neural network (VGGNet) for extracting features from WBC images and then filtering the resulting features using a statistically enhanced Salp Swarm Algorithm (SESSA) to extract only relevant and eliminate unnecessary features. The proposed hybrid approach performed very well in both accuracy and complexity reduction, which positively affects computation time and resource consumption. The SESSA optimization was successful in narrowing down the features number from 25 K to 1 K while improving performance at the same time. The results are the highest among all known published works on the same dataset, even compared to other convolutional network models. The combination of CNN feature extraction and SESSA feature optimization can be useful for solving other image classification tasks and machine learning optimization problems.

## Data Availability

All code and data required to reproduce the results are available  at https://go.uniwue.de/all-sessa.
